# Supplementary material for: Decoding network-mediated retinal response to electrical stimulation: implications for fidelity of prosthetic vision
Source: J Neural Eng. Author manuscript; Available in PMC 2022 Feb 3. (PMC8284336; doi:10.1088/1741-2552/abc535)
Supplement: Suppl Figure 1 [file NIHMS1718682-supplement-Suppl_Figure_1.pdf]

Supplemental Material

Supplemental Figure 1

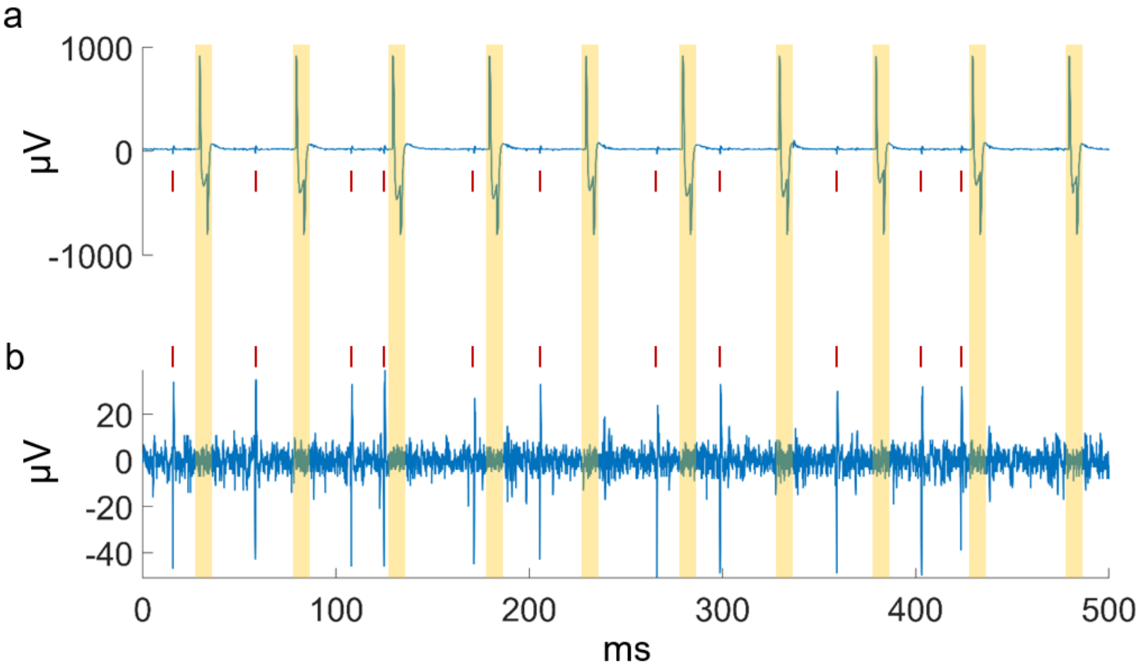

Example of a recording segment. (a) Unprocessed recording on one electrode of the multielectrode array (MEA). With 20 Hz electrical stimulation (yellow bars), we observe strong electrical artifacts at the recording electrodes. Segments of the recordings overlapping with the yellow bars were replaced with randomly generated noise ("blanking"). Periods of time shortly after the blanking were fitted and subtracted with a 7th-order polynomial. (b) Artifact-removed recording. Action potentials (red ticks) remain for spike sorting.
